# Visible Voices: Expressive arts with isolated seniors using trained volunteers

**DOI:** 10.1080/17533015.2013.817447

**Published:** 2013-08-01

**Authors:** Fay Wilkinson, Ann MacLeod, Mark W. Skinner, Heather Reid

**Affiliations:** a The Creative Cocoon, 2837 Haliburton Lake Road, P.O. Box 151, Eagle Lake, Ontario, Canada K0M 1M0; b Trent-Fleming School of Nursing, Trent University, 1600 West Bank Drive, Peterborough, Ontario, Canada K9J 7B8; c Department of Geography, Trent University, 1600 West Bank Drive, Peterborough, Ontario, Canada K9J 7B8; d U-Links Centre for Community-Based Research, P.O. Box 655, Minden, Ontario, Canada K0M 2K0

**Keywords:** Canada, expressive arts, participatory art, older people, volunteers, well-being

## Abstract

This practice-based paper describes an innovative program from Ontario, Canada that explored the potential for volunteer-facilitated expressive arts to contribute to the well-being of socially isolated rural seniors. Inspired by Arts on Prescription initiatives in the UK and coordinated by a Registered Expressive Arts Consultant/Educator, the program involved eight older volunteers and eight older participants engaged in a 10-week series of one-on-one intermodal art-making activities in the participants’ homes and institutional settings in 2009–2010. An evaluation of the program design and implementation is presented and the challenges and opportunities of expressive arts with isolated seniors using trained volunteers are discussed.

## Introduction

This practice-based paper describes an innovative program – *Visible Voices: Seniors Connecting Seniors through Expressive Arts* – that explored the potential for volunteer-facilitated expressive arts to contribute to the well-being of socially isolated rural seniors in Ontario, Canada. Founded in the early 1970s by McNiff, Knill and others at Lesley College Graduate School (USA), “expressive arts” as a field of practice has, until recently, been the domain of therapists. On the origin of the field, ISIS Canada (http://www.isis-canada.org) states,

(expressive arts) emphasized an interdisciplinary or ‘intermodal’ approach to the use of the arts for healing, in contrast to the specialized arts therapies, which at the time restricted practice to one artistic discipline and usually were based on an established psychological framework.

The International Expressive Arts Therapy Association (IEATA) (http://www.ieata.org) defines the field as, “[Combining] the visual arts, movement, drama, music, writing and other creative processes to foster deep personal growth and community development.” IEATA contributes to the legitimacy of expressive arts at the facilitator level by registering Expressive Arts Consultants/Educators, who are not necessarily artists and who are bound by a code of ethics and required to demonstrate, “Fluency in multi-modal expressive arts process: understanding how each arts modality deepens and builds on the other, and knowledge of how to choose modalities to best meet clients’ needs.” Expressive arts at the facilitator level, therefore, is related to participatory arts and the creative arts, all under the umbrella of “Arts and Health” (Clift et al., 2009; Cox et al., 2010; Sonke, Rollins, Brandman, & Graham-Pole, 2009; Wreford, 2010). The program profiled in this paper is an example of the growing range of expressive arts programs delivered at the facilitator level.

## Program Background

Visible Voices originated in 2008 in the form of a pilot program called Arts Rx (taking expressive arts to older adults one-on-one), which was inspired by Arts on Prescription initiatives in the UK (Stickley & Hui, 2012), and supported by the local Arts Council and Economic Development Corporation of a rural county in central Ontario. The pilot program was designed and delivered by the first author, in two long-term care facilities. In 2009–2010, as a result of the pilot’s success and funded by a New Horizons for Seniors grant from Human Resources and Development Canada, the expanded Visible Voices program was developed and implemented by the first author, a registered Expressive Arts Consultant/Educator, in collaboration with a local advisory committee and a group of older adult volunteers. Using trained volunteers, Visible Voices aimed to reach out into the community to socially isolated seniors using expressive arts as a means for expression, reflection, social engagement and overall well-being. The one-on-one, intermodal process at the heart of the program placed as much emphasis on the creative process as the final product, and no formal art training was needed for either the facilitator or the participant.

## Objectives and Rationale

The goal of Visible Voices was to enhance the life satisfaction of seniors who are engaged in volunteering as well as those senior participants who experience social isolation either living independently or in long-term care settings. The program objectives were (1) to train and enable older volunteers to engage in meaningful expressive arts activities with socially isolated seniors to enhance their own life-satisfaction; (2) to give isolated senior participants the opportunity to enhance their life-satisfaction through expressive arts activities and (3) to provide researchers the opportunity to understand the role of expressive arts experiences in relation to the challenges of aging.

In light of the growing focus on holistic approaches to aging in place that increasingly emphasize volunteer-based support and participation in volunteer activities as determinants of “successful” or “healthy” aging (Joseph & Skinner, 2012), the rationale for engaging older adult volunteers to facilitate art-making with socially isolated seniors in the context of their own home was three-fold. First, the program sought to improve the well-being of older participants in the community by maximizing their sense of control during a creative activity with a peer. Art-based participatory activities for older adults, for instance, have the potential to result in positive psychosocial and health outcomes (Castora-Binkley, Noelker, Prohaska, & Satariano, 2010). Second, the formal incorporation of volunteers in the program was aimed at providing an opportunity for older adults to reap the reported health benefits of participation in volunteerism (Gottlieb & Gillespie, 2008). Third, because professional resources are often scarce and difficult to access in rural areas, the use of volunteers was seen as a means of delivering outreach programs in underserviced contexts (Joseph & Skinner, 2012). Taken together, the potential for trained expressive arts volunteers, who are not necessarily artists, to enhance the well-being of older participants as well as their own health provided the rationale for the expanded program.

## The Program

All the experience in the world could not have prepared me for the amazing experience I shared with M. Life … is the skill set. Being in the moment, enjoying every opportunity that came our way. Being flexible, listening for the feelings as well as the information, celebrating moments, cups of tea and laughter. Just allowing the experience to flow and grow. This program is about being real … and growing together. (Volunteer)

Delivered in 2009/2010, the Visible Voices program featured a 1- to 2-h intermodal expressive arts experience, once a week for 10 weeks with volunteers working one-on-one at the participant’s home or long-term care residence. Under the leadership of the first author, the program involved eight older female volunteers (aged 55–75) and eight cognitively well, isolated seniors (two men and six women aged 65–95), six of whom were living in their homes and two in long-term care facilities. The volunteers included expressive arts facilitators who were graduates of the Fleming College Expressive Arts Post Diploma Program (http://www.flemingcollege.ca), an artist, a social worker, retired teachers, an infant mental health specialist and a nurse. For the most part, friends and family identified socially isolated seniors as potential participants. The program culminated in an afternoon of celebration bringing together the community, the participants and the volunteers. Written and informed consent to share artwork and narratives were obtained from all participants and volunteers. Careful attention was given to the ethical obligations of working with vulnerable participants, including obtaining confidentiality agreements and police checks from the volunteers involved as well as ensuring the anonymity of the isolated older adults by omitting identifying information in the evaluation presented herein. Ethics approval for the project was obtained from Trent University Research Ethics Board.

## Training and Supporting Volunteers

**Figure 1:**
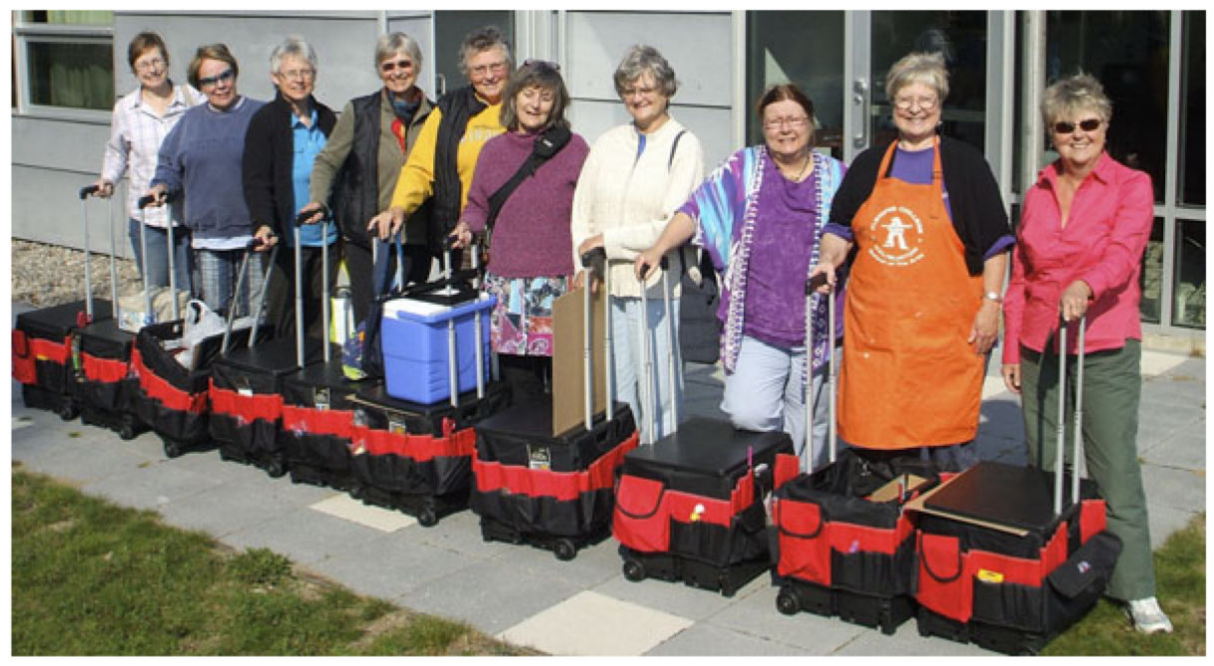
Volunteers with Their Art Carts.

Volunteers were recruited by the program leader primarily by word-of-mouth and were paired with participants based on their interests and backgrounds. The initial 15-h training included a program orientation and expressive arts facilitation (the use of materials, intermodal transitions using different mediums, as much emphasis on the creative process as the final product, not being attached to an outcome and the effective use of metaphors). This differentiated expressive arts from art lessons and crafts. Ways to provide a safe space where the volunteer–participant pairs could create honestly and build trust were covered as were other hallmarks of expressive arts experiences (participant-centred, non-judgmental, non-interpretative and playful). Of the ten trained volunteers, eight chose to continue with the delivery of the program (Figure 1).

**Figure 2:**
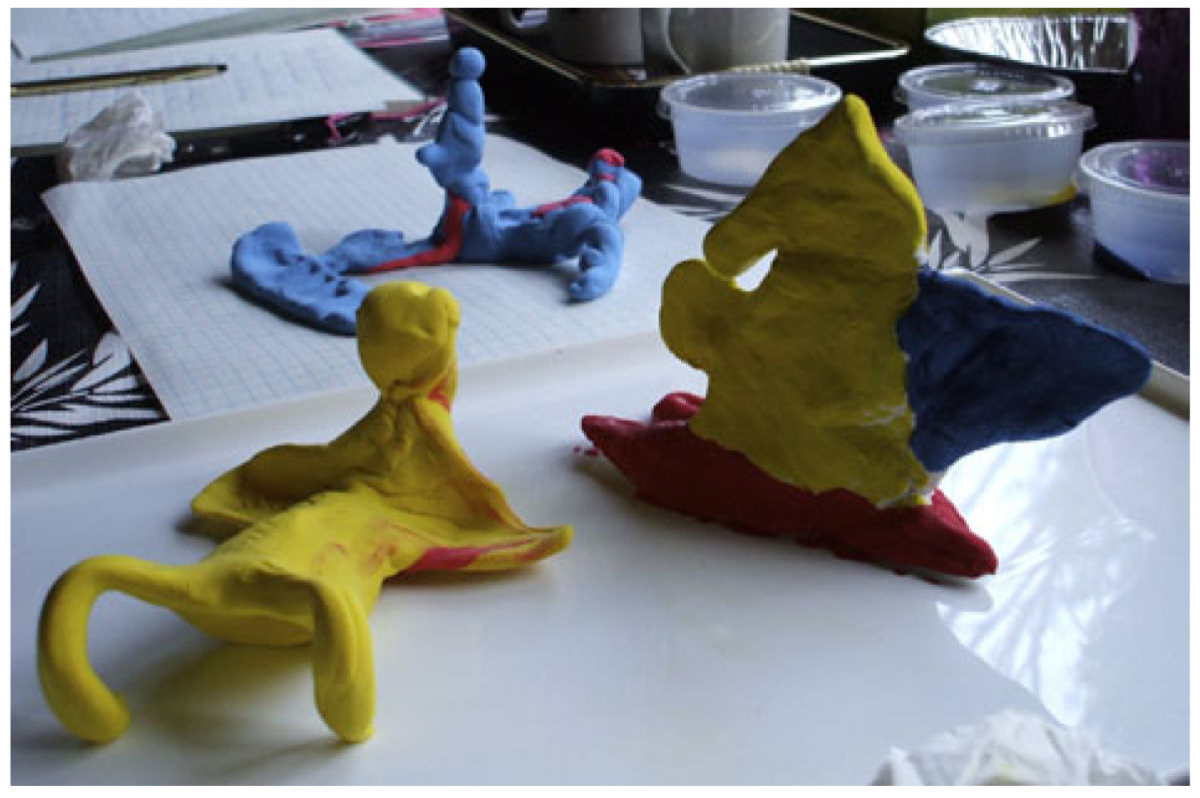
Co-created Sculptures that Led to Story.

Once paired with a participant by the program leader, the volunteers undertook a series of 10 in-person sessions, which were not prescribed either in terms of which expressive arts activity or what art-making materials they were to use. Volunteers were encouraged to tailor the sessions, create art alongside their participant (if it was appropriate) and to reflect on their experience through journaling and art-making (Figure 2).

The program leader facilitated four group sessions to provide support and debrief volunteer experiences offering methods to facilitate expressive arts that would meet the needs of both the participant and the volunteer. Guidance on fostering the relationship, establishing boundaries, focusing on the art-making, and anticipating closure were discussed. These sessions brought the training hours up to 25 and were well received by the volunteers, as noted below:

The meetings gave me valuable information about what projects to pursue both in practical experience and written material. It was great to be in the presence of other volunteers and have the experience of being part of a group doing such valuable work. (Volunteer)

**Figure 3:**
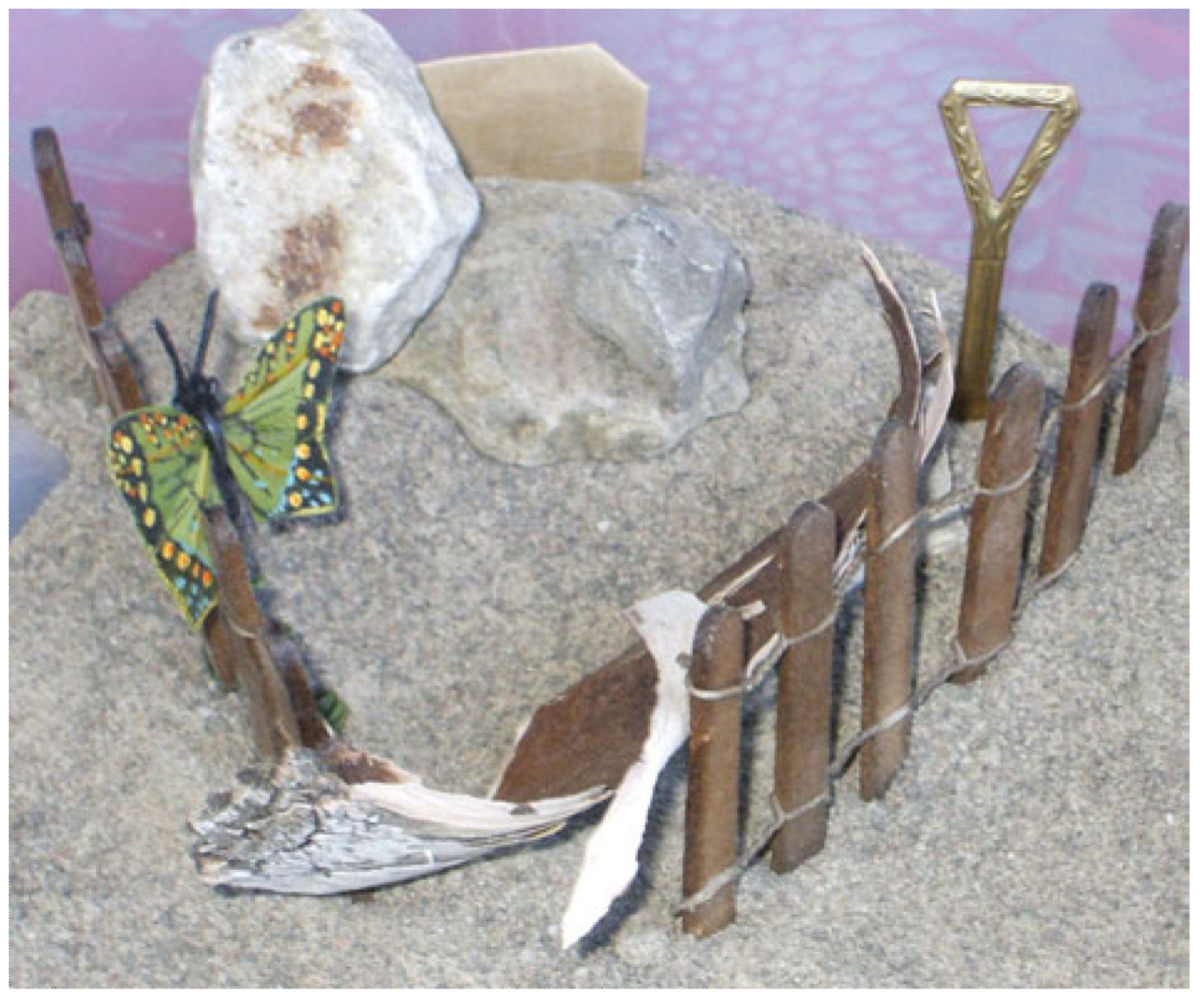
Detail of Volunteer Group Sandtray.

A group sandtray activity was used in the final debriefing session with the volunteers, which allowed them to explore and articulate their overall experience (Figure 3).

## Program Evaluation

To evaluate the program, data was collected from the leader’s field notes, volunteers’ weekly logs, photographs of work created by the older adult participants and volunteers, transcripts of the four volunteer debriefing meetings, and program evaluation questionnaires completed by the volunteers and participants. A full analysis of these data as they relate to the potential contribution to art-based research on the role of expressive arts in relation to the challenges of aging (i.e. the third program objective) is beyond the scope of this practice-based paper, but is underway elsewhere (see MacLeod, Skinner, Wilkinson, & Reid, under review). For the purpose of this descriptive paper, general insights related to the first and second program objectives are outlined.

**Figure 4:**
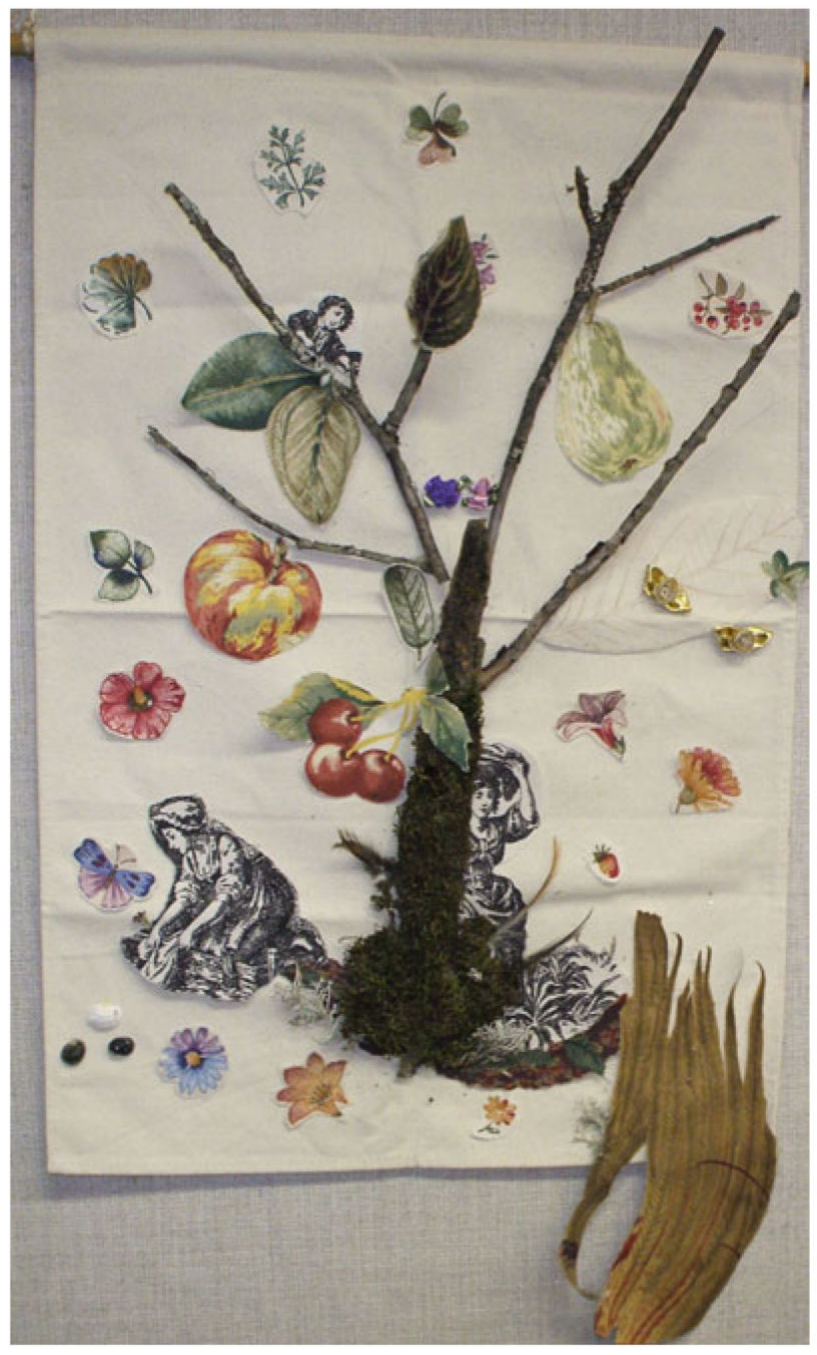
A Participant’s Metaphorical Tree of Life.

Feedback on the first objective, to train and enable older volunteers to engage in meaningful expressive arts activities with socially isolated seniors to enhance their own life-satisfaction, was taken from volunteer evaluations. When asked, “Has Visible Voices made any difference to you personally?” the responses show an opportunity to reflect on their own lives, learning about the aging process, doors opened to new perceptions and increased confidence. For instance, the volunteer responses include: “It has deepened my understanding of the aging process and how art-making can play a role” and “This program touched me very deeply. In exploring my participant’s Tree of Life artwork with her, I gained a new perception of my own life.” The volunteers, as a result of co-creating with their participant and reflecting on their experience, as in the Tree of Life example, created significant meaning from the opportunity (Figure 4).

**Figure 5:**
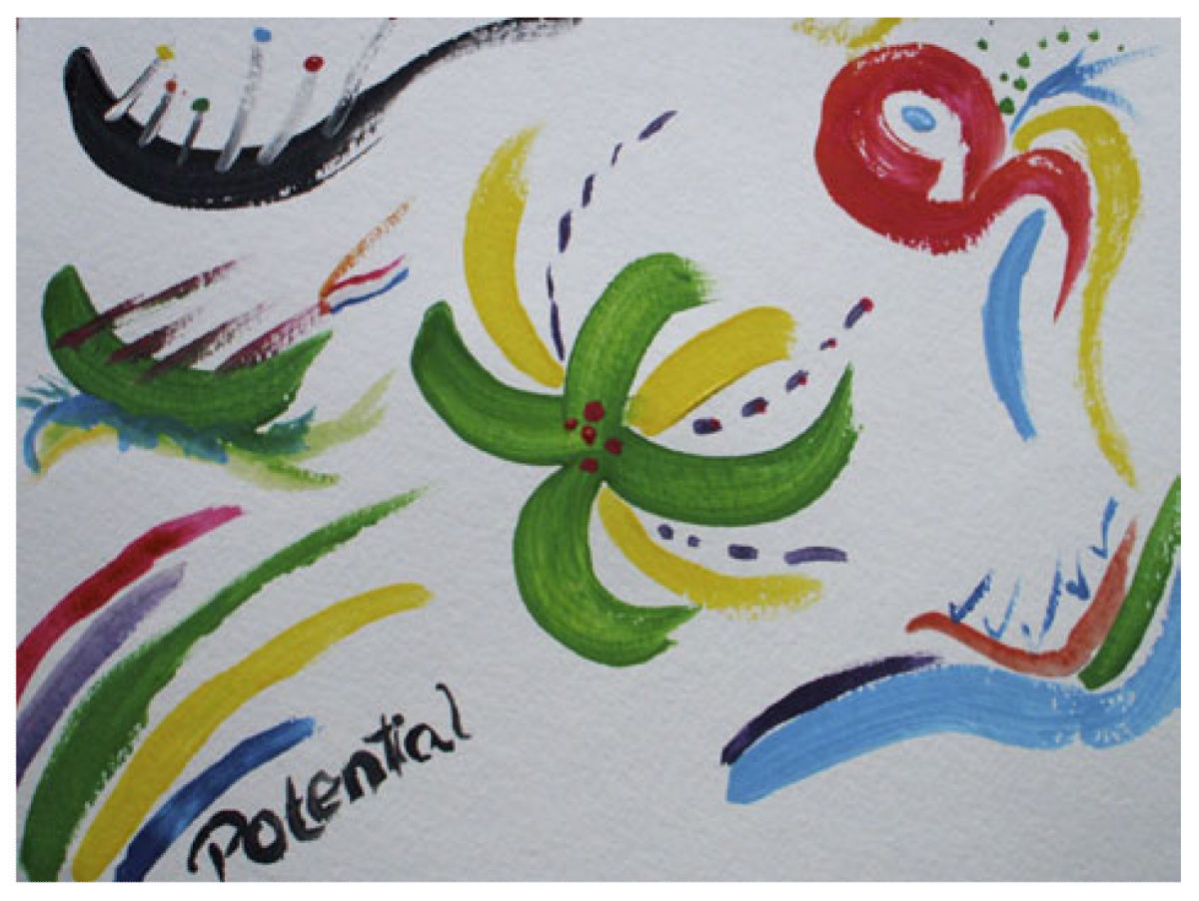
“Potential” Painted by a Participant.

Participant evaluations spoke to the second objective, to give isolated senior participants the opportunity to enhance their life-satisfaction through the expressive arts process. When asked to, “Describe what Visible Voices is to them,” participant responses included: “It was very good. I would say it is an effort to tap possibly unknown potential,” “It gives people a chance to express themselves” and “This is an excellent program. It lets it be you, something to look forward to weekly. It has brought out my inner-self.” Indeed, all of the participants would recommend the program to others and would participate again. As one older participant stated poignantly, “I wish it would last forever!” (Figure 5).

## Reflections on the Program

Challenges and solutions were discussed at group debriefing meetings as well as between the program leader and each volunteer individually. Although using volunteers as expressive arts facilitators expanded the reach and scope of expressive arts experiences to socially isolated adults, things can go awry. For example, a clash of values, expectations not realized, personal boundaries crossed, and the physical environment that interfered with the creative process and the volunteer’s sense of well-being in the space. Prompt phone access to the program leader was important as well as the regular volunteer debriefing sessions.

The volunteers who used their own reflective art-making post-session to work through the challenges they were facing with their participants found it to be a valuable exercise. As one volunteer noted, “I do think that the reflective part for me was very therapeutic.” Another volunteer created collages then shifted modalities into poetry to help her process a difficult session:

Uncomfortable, demanding boulders
Challenge uphill climb
Proud crippled willing player
Creates the momentum
To move from darkness to light

The poem highlights challenges for the volunteer to maintain a personal connection while protecting personal boundaries.

Other challenges included logistical issues. For example, volunteers commented, “The physical surroundings (were) extremely cluttered with very little space to work” and “It was difficult when her husband was in the same space while we were working.”

Volunteers also found their own creative solutions by being persistent. They cited the importance of having empathy and making it fun as well as needing to change their expectations. Two volunteer comments included: “I showed compassion and concern and tried always to make what we were doing interesting for her and fun” and “I realized I was not there to fix/clean up, but to do art. Our next visit together was congenial and productive.” Each volunteer, to a greater or lesser extent, was comfortable with ambiguity, and was able to use the program leader for support and as a resource.

From the perspective of the program leader, designing a training program that gave “just enough” information for volunteers from diverse backgrounds was challenging. That said, the relationship was emphasized as being one of the most important elements of the work and the need to be flexible was essential; that is, to have a plan, but be prepared to throw away the plan if necessary in order to serve the participant effectively.

The comfort, experience, skill level and personal attributes of the volunteer influenced how expressive art was facilitated with their respective participants. The more experienced expressive arts facilitators moved between modalities when the opportunity arose resulting in deeper conversations around created pieces, as demonstrated in the logs. Volunteers from other backgrounds for the most part stayed with one modality in each session, which resulted in appreciated social experiences with less personal reflection. Regardless of experience, upon completion of the program, seven of the eight volunteers were interested in continuing their commitment.

## Next Steps

Understanding further the role of trained volunteers as expressive arts facilitators would be useful for expanding the edge of expressive arts practice, particularly as it relates to vulnerable older people in under-serviced areas. The Visible Voices program offers an example of a way of working with expressive arts trained facilitators, in this case volunteers, who are sensitive to the needs of the population and understand the intermodal nature of the work. Based on our experience with this program, we contend that there needs to be a trained and experienced expressive arts practitioner who underpins the program and supports the volunteers working with expressive arts in this context. More broadly, given the emerging body of research that demonstrates the power of art-making to enhance well-being and social engagement, expressive arts practitioners and trained volunteers have an important, yet under-researched, role to play in addressing mental health promotion of the older socially isolated adults (Moody & Phinney, 2012). One interesting avenue for further research would be to explore the impact on younger trained volunteers working with seniors as compared to older adult volunteers. However we are mindful that, in rural areas in particular, the majority of volunteers are older (Joseph & Skinner, 2012).

The Visible Voices program has taught us a lot about enhancing volunteer-led expressive arts programs. Moving forward, the following key lessons need to be incorporated into any future program planning and implementation. First is the need for careful attention to volunteer safety, including the risks of driving to remote locations, working in older adults’ homes and the importance of telephone support by the program leader as reassurance to address both physical and emotional safety. Second is the related need for careful attention to the sensitivities of vulnerable participants, including their consent, privacy, and safety, as well as the particular issue of pairing volunteers with participants in the close-knit rural communities, where there needs to be an awareness of past and present relationships. Third, in future programs, training needs to incorporate suggestions from the volunteers, specifically, more time with the art cart contents, more information regarding expressive arts and using intermodal shifts, more role plays, clear volunteer responsibilities around reporting living conditions, and/or physical complaints and personal boundary issues. Finally, there is a further need to consider the value and drawbacks to concurrent art-making.

With these lessons in mind, the next version of Visible Voices is already underway - *Visible Voices: Through the Looking Glass* – which aims to explore depression with seniors through expressive arts and engage the community through presentations and reflective art-making (see Wilkinson, 2013).

## References

[R1] Castora-Binkley M, Noelker L, Prohaska T, Satariano W (2010). Impact of arts participation on health outcomes for older adults. Journal of Aging, Humanities and the Arts.

[R2] Clift S, Camic P, Chapman B, Clayton G, Daykin N, Eades G, White M (2009). The state of arts and health in England. Arts & Health: An International Journal for Research, Policy and Practice.

[R3] Cox S, Lafreniere D, Brett-MacLean P, Collie K, Cooley N, Dunbrack J, Frager G (2010). Tipping the iceberg? The state of arts and health in Canada. Arts & Health: An International Journal for Research, Policy and Practice.

[R4] Gottlieb B, Gillespie A (2008). Volunteerism, health, and civic engagement among older adults. Canadian Journal on Aging.

[R5] Joseph AE, Skinner MW (2012). Voluntarism as a mediator of the experience of growing old in evolving rural places and changing rural spaces. Journal of Rural Studies.

[R6] MacLeod A, Skinner MW, Wilkinson F, Reid H Connecting socially isolated older rural adults through expressive arts with older volunteers. Canadian Journal on Aging.

[R7] Moody E, Phinney A (2012). A community-engaged art program for older people: Fostering social inclusion. Canadian Journal on Aging.

[R8] Sonke J, Rollins J, Brandman J, Graham-Pole J (2009). The state of the arts in healthcare in the United States. Arts & Health: An International Journal for Research, Policy and Practice.

[R9] Stickley T, Hui A (2012). Social prescribing through arts on prescription in a UK city: Participants’ perspectives. Public Health.

[R10] Wilkinson F (2013). Visible Voices: Through the looking glass [Video presentation].

[R11] Wreford G (2010). The state of the arts and health in Australia. Arts & Health: An International Journal for Research, Policy and Practice.

